# The gut microbiome’s role in the development, maintenance, and outcomes of sepsis

**DOI:** 10.1186/s13054-020-02989-1

**Published:** 2020-06-01

**Authors:** Max W. Adelman, Michael H. Woodworth, Charles Langelier, Lindsay M. Busch, Jordan A. Kempker, Colleen S. Kraft, Greg S. Martin

**Affiliations:** 1 Division of Infectious Diseases, Department of Medicine, Emory University School of Medicine, 49 Jesse Hill Jr. Drive, Atlanta, GA 30303 USA; 2 Division of Infectious Diseases, Department of Medicine, University of California, San Francisco, CA USA; 3 Critical Care Medicine Department, National Institutes of Health Clinical Center, Bethesda, MD USA; 4 Division of Pulmonary, Allergy, Critical Care and Sleep Medicine, Department of Medicine, Emory University School of Medicine, Atlanta, GA USA; 5 Department of Pathology and Laboratory Medicine, Emory University School of Medicine, Atlanta, GA USA; 6 Emory Critical Care Center, Emory Healthcare, Atlanta, GA USA

**Keywords:** Sepsis, Gut microbiome, Review, Probiotics, Fecal microbiota transplant

## Abstract

The gut microbiome regulates a number of homeostatic mechanisms in the healthy host including immune function and gut barrier protection. Loss of normal gut microbial structure and function has been associated with diseases as diverse as *Clostridioides difficile* infection, asthma, and epilepsy. Recent evidence has also demonstrated a link between the gut microbiome and sepsis. In this review, we focus on three key areas of the interaction between the gut microbiome and sepsis. First, prior to sepsis onset, gut microbiome alteration increases sepsis susceptibility through several mechanisms, including (a) allowing for expansion of pathogenic intestinal bacteria, (b) priming the immune system for a robust pro-inflammatory response, and (c) decreasing production of beneficial microbial products such as short-chain fatty acids. Second, once sepsis is established, gut microbiome disruption worsens and increases susceptibility to end-organ dysfunction. Third, there is limited evidence that microbiome-based therapeutics, including probiotics and selective digestive decontamination, may decrease sepsis risk and improve sepsis outcomes in select patient populations, but concerns about safety have limited uptake. Case reports of a different microbiome-based therapy, fecal microbiota transplantation, have shown correlation with gut microbial structure restoration and decreased inflammatory response, but these results require further validation. While much of the evidence linking the gut microbiome and sepsis has been established in pre-clinical studies, clinical evidence is lacking in many areas. To address this, we outline a potential research agenda for further investigating the interaction between the gut microbiome and sepsis.

## Background

Sepsis, a dysregulated immune response to infection resulting in end-organ damage and potentially death, is a major public health threat [1]. Sepsis affects 1.7 million patients in the USA annually with mortality of up to 50% [2, 3]. Despite high incidence, morbidity, and mortality, there are few established treatments for sepsis. The mainstays of therapy—antibiotics and supportive care—have not changed significantly for decades.

The gut microbiome modulates several responses to sepsis and is a potential therapeutic target in sepsis [4]. Loss of normal gut microbiome structure and function has been implicated in several diseases including *Clostridioides difficile* infection (CDI), inflammatory bowel disease, and obesity [5]. While the pathogenesis of sepsis is multifactorial and incompletely understood, there is increasing evidence that gut microbiome disruption predisposes to sepsis and negatively impact sepsis outcomes [6]. In this review, we highlight this evidence, review the use of fecal microbiota transplant (FMT) in sepsis, and outline research priorities for clarifying the role of the gut microbiome in sepsis. Although research into associations between the gut microbiome and sepsis has been increasing, differences in methods and outcome measures make comparisons across studies difficult. Table 1 provides an overview of important terminology for interpreting microbiome research and key terms used in this review.

**Table 1:** Glossary of key concepts for interpreting microbiome research

Concept	Definition	Notes
Techniques for identifying microbiome components		
Culture-based	Uses traditional techniques for culturing bacteria to determine which species are present	Some species (e.g., anaerobes) are difficult to culture; once cultured, definitive identification may be difficult
16S rRNA sequencing	Uses a conserved region of bacterial RNA to identify bacteria, combined with a species-specific sequence to determine which species are present	Unable to identify genes or presence of non-bacterial components (e.g., protozoa or fungi)
Metagenomic sequencing	Uses “unbiased” sequencing to determine all genes present in a sample and construct community structure; allows for determination of community composition and function	Remains relatively expensive, although cost has decreased; applications are still most suitable for research
Classification of microbiome composition		
Abundance	Relative amount of specific bacterial groups in a sample	Most techniques only allow for determination of relative abundance of bacteria, not absolute (i.e., unable to determine total number of bacteria present in a sample)
α-diversity	Within-group microbiome diversity	Describes the makeup of a microbial community from one sample (e.g., one patient or one body site)
β-diversity	Between-group microbiome diversity	Allows for comparisons between groups of samples
Dysbiosis	Describes a microbial community that has been altered from its normal structure	Can be nonspecific; for example, unclear if this refers to decreased relative abundance of one group, decreased α-diversity, or another measure

## Gut microbiome disruption predisposes to sepsis

Although thought to be sterile in utero*,* the neonatal intestine is colonized at birth. Over the first several weeks of life, acquisition of phylogenetic diversity, especially with obligate anaerobes, protects from pathogen colonization [7, 8]. A 2019 prospective cohort study of over 200 preterm infants found that increased bacterial diversity and anaerobic bacterial colonization of the neonatal gut microbiome protects against sepsis [9]. Importantly, bacterial species that predominate in the absence of anaerobes, including *Staphylococcus* species and *Escherichia coli*, are those that translocate and cause bacteremia [7, 9, 10].

Less is known about gut microbiome changes that predispose adults to sepsis, but an early study demonstrated a pathogenic role of gut microbiome colonization and translocation in post-operative sepsis [11]. There are few studies that prospectively track gut microbiome changes prior to sepsis onset, and all have only been published as conference abstracts to date [12–14]. However, these preliminary studies indicate that patients with low gut microbiome diversity [12, 14] and high relative abundance of pathogenic gram negatives and enterococci [13] are at higher risk of sepsis. Other studies have shown similar risk factors for bacteremia in hematopoietic stem cell transplant recipients [15, 16] and for infection in intensive care unit (ICU) patients [17].

Two recent large epidemiologic studies provide circumstantial evidence that gut microbiome disruption is a risk factor for sepsis [18, 19]. Although neither of these studies specifically characterized the gut microbiomes of included patients, they examined the impact of CDI and broad-spectrum antibiotics on sepsis development. Because CDI and broad-spectrum antibiotics are closely linked with gut microbiome disruption, patients who had CDI or received broad-spectrum antibiotics should have more significant microbiome disruption than those who did not. The first, a 2015 study of over 10,000 Medicare recipients, examined the risk of sepsis after an episode of CDI [18]. In this cohort, patients were 70% more likely to develop severe sepsis after hospitalization for CDI than after hospitalization for an infectious cause other than CDI. The second study looked at risk of severe sepsis after receipt of different classes of antibiotics in over 12 million patients [19]. Patients who received antibiotics most associated with CDI (e.g., 3rd and 4th generation cephalosporins, carbapenems, and fluoroquinolones) had 65% higher odds of readmission for severe sepsis or septic shock than patients who did not receive antibiotics. This association was attenuated in patients who received antibiotics less associated with CDI (e.g., 1st and 2nd generation cephalosporins and macrolides). These studies therefore suggest a strong correlation between gut microbiome disruption and subsequent sepsis development. Although the correlation may be strong, the mechanisms by which these microbiome changes affect sepsis risk, and potential confounders of this risk, were not evaluated in these studies and require clarification.

In addition to predisposing to sepsis, decreased gut microbiome diversity modulates host response to sepsis in animal models. In one experiment, genetically identical mice purchased from different vendors had compositionally distinct gut microbial communities (i.e., β-diversity) and different amounts of diversity (i.e., α-diversity). When subjected to experimental abdominal sepsis, mice with greater α-diversity were more likely to survive than those with lower α-diversity (47% vs. 10% 7-day survival). Co-housing mice led to equilibration of both α- and β-diversity between groups with resultant improved sepsis survival among mice previously more likely to die [20]. Similarly, the gut microbiomes of mice who survived sepsis were protective against sepsis when given to sepsis-susceptible mice via FMT [21]. Similar effects of microbiome depletion and loss of diversity on mortality have been shown in several experimental models of sepsis [22, 23], including models of influenza A and pneumococcal pneumonia [24, 25]. Potential mechanistic links between gut microbiome diversity and sepsis susceptibility are discussed in the next section.

Whether gut microbiome disruption prior to sepsis onset worsens sepsis outcomes in humans remains unknown. One randomized controlled trial (RCT) examined the impact of pre-treatment with broad-spectrum antibiotics on outcomes in healthy young men given intravenous lipopolysaccharide (LPS) [26]. Although subjects randomized to broad-spectrum antibiotics had decreased α-diversity and lower abundance of several beneficial gut bacteria, there was no effect on surrogate markers of sepsis severity including vital signs and fibrinolysis [26]. As this study shows, gut microbiome composition and diversity are unlikely to account for all of the clinical heterogeneity seen in sepsis. The gut microbiome is likely one of many factors that regulate systemic sepsis response; more research is needed to clarify interactions between regulatory mechanisms.

## Mechanisms of increased sepsis susceptibility

### Selection for pathobionts

In the presence of protective commensal bacteria, bacteria with pathogenic potential that reside in the intestinal lumen of healthy hosts (“pathobionts”) may not be able to proliferate and cause disease [27]. Loss of protective bacterial taxa allows for pathobiont proliferation [28, 29]. In a key early study, when mice were exposed to colonic inflammation and antibiotics, the gut microbiome was characterized by expansion of a pathogenic clone of multi-drug resistant (MDR) *E. coli*, which disseminated systemically [30].

In a separate experiment, mice were fed a high- or normal-fat diet, given broad-spectrum antibiotics, and then subjected to partial hepatectomy. The high-fat diet mice had decreased gut microbiome α-diversity, were less able to survive partial hepatectomy, and had expansion of MDR gram-negative bacteria compared to mice fed a normal diet [31]. High-fat diet mice had higher mortality and more bacterial dissemination from the gut, demonstrating that decreased gut microbiome diversity can predispose to intestinal translocation even if the primary injury is remote.

### Altered immune response

The microbiome’s influence on immune development begins with colonization of the neonatal gastrointestinal tract at birth [7, 32]. Germ-free neonatal mice have decreased development of bone marrow myeloid precursors and resultant decreased myeloid lineage cells in the spleen, rendering them susceptible to *E. coli*, *Listeria monocytogenes*, and *Staphylococcus aureus* sepsis [33, 34]. Intestinal re-colonization can reduce immunologic dysfunction that predisposes to increased sepsis susceptibility [34].

After intestinal colonization during the neonatal period, differences in gut microbiome composition direct differential immune responses to sepsis. Mice with increased gut microbiome α-diversity have improved sepsis survival, which is mediated by a distinct immunophenotype characterized by an increased CD4+ T cell response [20]. Enhancing microbiome α-diversity in mice by co-housing not only led to increased sepsis survival, but also changed the immune response to sepsis [20]. Mice who survived sepsis had improved outcomes when exposed to sepsis with similar bacteria as those that colonized their intestines, due to improved T cell response against those specific bacterial antigens [35]. In addition to impacting T cell responses, the gut microbiome also influences humoral immunity: commensal bacteria direct IgA production, which is protective in subsequent sepsis through bacterial homology [36]. These studies indicate a role for the gut microbiome in “priming” the immune system to respond to sepsis.

After sepsis onset, gut microbiome changes characterized by decreased abundance of gut commensals affect inflammatory responses. Animal studies have demonstrated conflicting results regarding specific inflammatory pathways, reflecting the complexity of the relationship between the gut microbiome and the immune system. For example, in a mouse model of *Streptococcus pneumoniae* sepsis, pre-treatment with oral antibiotics prior to sepsis onset was associated with lower levels of lung TNF-α, a pro-inflammatory cytokine [25], whereas others have shown the opposite effect of gut microbiome depletion on TNF-α [21, 22, 37–39]. Despite differences in specific cytokine expression between studies, the overall effect of alteration of normal gut microbiome structure prior to sepsis onset appears to be a more robust inflammatory response to sepsis [21, 22, 25, 37–41].

Differences in cytokine expression may be due to the effect of commensal bacteria on specific immune pathways. A study of 500 healthy adults demonstrated interactions between gut microbial commensal species and cytokine expression by correlating gut microbiome composition with cytokine response when peripheral blood mononuclear cells were stimulated by microbial antigens ex vivo [42]. For example, the authors showed that the commensal bacteria *Coprococcus comes* influences production of cytokines IL-1β and IL-6 to modulate the acute inflammatory response to *Candida albicans* infection [42].

While important, ex vivo studies do not fully replicate conditions present during sepsis in humans. Outside of a few small studies, there is little human data on the gut microbiome’s impact on immune modulation in sepsis [26, 43–46]. One study did not find a relationship between the gut microbiome and immune response in experimentally induced sepsis [26], whereas other studies in clinical sepsis have shown a link between gut microbiome alterations characterized by an increase in pathobionts and an exuberant immune response [43–45]. Given the importance of immune dysregulation in sepsis pathogenesis [47], the gut microbiome’s role in immune response to sepsis deserves further study.

### Decreased production of beneficial microbial products

Commensal members of the gut microbiome produce short-chain fatty acids (SCFAs) which regulate several functions of the gut microenvironment [48]. For example, Clostridia and *Faecalibacterium* produce the SCFA butyrate, which influences colonic regulatory T cell differentiation through upregulation of *Foxp3,* a key regulatory T cell transcription factor [49], and inhibits histone deacetylation to decrease NF-κB-regulated pro-inflammatory cytokines, including TNF-α and IL-6 [50].

In addition to their immune effects, SCFAs are crucial for epithelial cell function [51]. Acetate, produced by the gut commensal *Bifidobacterium*, protected mice from intestinal *E. coli* translocation through its effect on epithelial cell function [52]. Epithelial cell metabolism of butyrate decreases tissue oxygen concentration, thereby stabilizing HIF-1, a transcription factor that regulates several genes important for barrier function [53]. Preliminary data indicate that lower abundance of butyrate-producing bacteria may be a risk factor for sepsis onset [12], and increased gut membrane permeability is one plausible explanation that requires validation. For example, elevated serum markers of gut permeability including zonulin and FABP2 are associated with a gut microbiome with predominant gram negatives [54], but if these markers are elevated prior to sepsis onset in association with microbiome disruption is unknown.

## Sepsis worsens gut microbiome disruption

**Fig. 1:**
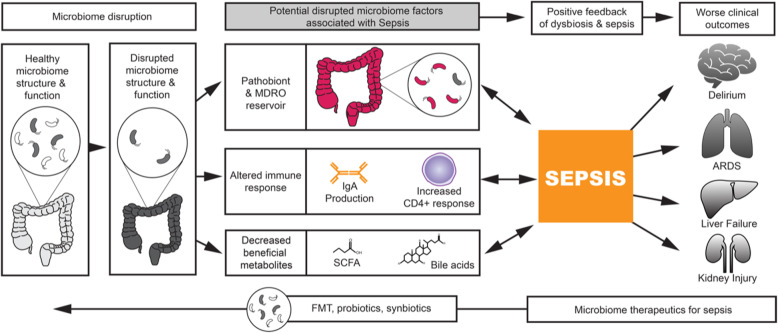
Conceptual illustration of the pathway between gut microbiome disruption and sepsis. Gut microbiome alteration predisposes to selection for pathobionts (potentially pathogenic bacteria that can reside in the gut microbiome), immune dysregulation, and decreased production of short-chain fatty acids, beneficial products produced by the gut microbiome. These changes can lower the threshold for sepsis development. Sepsis and treatment with antibiotics can drive worsening microbiome disruption in a vicious cycle, contributing to ongoing end-organ dysfunction. ARDS, acute respiratory distress syndrome; FMT, fecal microbiota transplant; MDRO, multi-drug resistant organism; SCFA, short-chain fatty acid

As described above, alterations in the gut microbiome can predispose to sepsis by allowing for proliferation of pathobionts, promoting a dysregulated immune response, and decreasing production of beneficial SCFAs. Gut microbiome changes not only affect outcomes prior to sepsis onset: after sepsis onset, alterations in normal gut microbiome structure can worsen and contribute to worse outcomes (Fig. 1).

The mechanisms underlying sepsis-driven gut microbiome changes are unclear. Antibiotics are a major driver of sepsis-related microbiome alteration [55]. However, opioids [56], parenteral nutrition [57], and proton-pump inhibitors [58] also have an effect. Clearly, the benefits of these interventions likely outweigh their deleterious microbiome impacts. An understanding of the impact of critical illness and associated interventions on the gut microbiome is necessary to inform research to limit these adverse effects.

Several prospective cohort studies have identified an association between decreased gut microbiome diversity in sepsis and higher relative abundance of potentially pathogenic bacteria including aerobic gram negatives [39, 46, 56, 59–61]. The earliest study to examine microbiome changes in critically ill patients showed that patients with systemic inflammatory response syndrome (SIRS) had lower abundance of obligate anaerobes, and their gut microbiomes were enriched with potential pathogens such as *Staphylococcus* and *Pseudomonas* [46]. As expected, a significant number of these patients were being treated with antibiotics, which confounded the relationship between sepsis and observed microbiome changes.

Early studies relied on culture-based techniques for determination of microbiome composition (Table 1). With increasing use of culture-independent techniques including 16S ribosomal RNA (rRNA) and metagenomic next-generation sequencing, recent studies have detected small changes in abundance of difficult-to-culture bacterial commensals that may impact sepsis outcomes. For example, *Faecalibacterium*, which is associated with pathways that decrease intestinal inflammation, generally occupies a small niche in the gut microbiome and is wiped out during sepsis [59, 60]. The disappearance of *Faecalibacterium* and other commensals allows hospital-acquired pathogens such as *Enterococcus* to colonize the intestinal microbiome during sepsis [62]. In turn, the intestinal microbiome acts as a reservoir for MDR organisms [28, 56]. These colonizing pathogens may disseminate and cause bacteremia later in the course of sepsis [46, 63].

In addition to allowing domination by organisms with significant pathogenic potential, alterations in microbiome function worsen detrimental changes seen prior to sepsis onset. For example, patients with sepsis have fewer fecal SCFAs [46, 64], which may worsen gut epithelial integrity and immune dysfunction seen in sepsis [4, 47]. No studies have tracked longitudinal microbiome changes in sepsis survivors after discharge from the hospital to determine how long these microbiome changes persist. However, preliminary studies indicate that decreased SCFA concentrations last through at least 6 weeks of hospitalization, corresponding with persistent pathobiont colonization [64].

## Gut microbiome and end-organ damage in sepsis

Sepsis affects end-organ damage through several mechanisms, including alterations in gut microbiome structure and function. Gut microbiome-derived SCFAs protected against acute kidney injury (AKI) in a mouse model of sepsis [65] and a specific gut microbiome composition decreased liver injury in a separate experiment [21]. Fascinatingly, the gut microbiome appears to impact delirium not only indirectly via inflammatory pathways, but also by bacterial translocation to the brains of septic patients with gut-derived bacteria [41, 44]. In an animal experiment on sepsis-induced delirium, mice with sepsis had decreased microbiome diversity and more severe neurocognitive damage, including more seizures, and FMT lessened the detrimental neurologic impact [41].

A relationship between the gut and lung microbiome has been established by several key studies but still needs to be translated to patients with sepsis. In one study, the lung microbiome of mice with non-pulmonary sepsis was enriched with gut bacteria, including *Enterococcus*, even though the mice did not have clinical bacteremia or pneumonia [66]. Importantly, this was true whether sepsis was induced via an intestinal puncture model or through systemic LPS injection, indicating that systemic inflammation was sufficient to affect end-organ function and microbiome composition.

In humans, patients with the acute respiratory distress syndrome (ARDS) have significantly higher abundance of enteric bacteria in their lung microbiomes than do healthy controls, and this correlates with higher markers of systemic inflammation [66]. Although not specific to sepsis, gut microbiome changes in hematopoietic stem cell recipients precipitated pulmonary complications, and in one patient, intestinal expansion of *Klebsiella pneumoniae* closely predated *K. pneumoniae* pneumonia [67]. One plausible explanation for this is the “gut-lymph theory,” by which increased intestinal permeability leads to bacterial translocation to lymphatics and ultimately the pulmonary circulation via the thoracic duct [68].

Despite increasing evidence that the gut microbiome affects end-organ damage in non-human studies and in diseases other than sepsis, only one small study in humans has looked at whether gut microbiome features (including decreased diversity and changes in composition) are associated with end-organ dysfunction in sepsis, including AKI and ARDS, and did not identify an effect on these outcomes [59]. Gut microbiome composition with fewer total obligate anaerobes [63] and abundant *Enterococcus* [17] at sepsis onset have been associated with mortality, whereas other studies have shown that microbiome diversity does not appear to be associated with mortality in sepsis [17, 59, 61]. In some of these studies, not all patients had sepsis, and sepsis patients were not analyzed as a subset [17, 59, 61]. Further studies designed to specifically track adverse outcomes in sepsis are needed to characterize the impact of gut microbiome changes, both composition and diversity, on sepsis outcomes.

## Microbiome-based therapeutics

### Selective digestive decontamination, probiotics and synbiotics

The past 30 years have seen increasing interest in targeting the gut microbiome to improve outcomes of critically ill patients. One microbiome-related approach to preventing sepsis from MDR bacteria has involved “selective” oral or digestive decontamination using oral or parenteral antimicrobials with theoretical activity against many resistant healthcare-associated pathogens [69, 70]. Although some data do not show significant long-term effects of these strategies [71], concern for re-colonization with MDR bacteria soon after treatment discontinuation and undesired selection for even more resistant organisms in the microbiome has limited uptake of selective oral and digestive decontamination [70, 72].

Probiotics, i.e., bacteria associated with potential beneficial properties, are perhaps the most studied microbiome-based intervention to prevent sepsis and improve sepsis outcomes. A 2017 RCT of over 4500 healthy newborns in rural India found that a synbiotic (i.e., a probiotic combined with a non-digestible compound that the probiotic requires for metabolism), in this case, *Lactobacillus plantarum* and fructooligosaccharide, reduced neonatal sepsis or death by 40% [73]. Whether these results are generalizable to regions with fewer nutritional deficiencies or infants at higher risk is uncertain.

Furthermore, the effect of specific probiotic species and strategies to ensure probiotic colonization require investigation. A RCT of the probiotic *Bifidobacterium breve* as prophylaxis in over 1000 very preterm infants at high risk of sepsis did not demonstrate reduction in sepsis incidence or mortality compared to placebo [74]. A sub-analysis using 16S rRNA sequencing showed that probiotic administration was not associated with microbiome composition or α-diversity, and only 70% of infants who received the probiotic were colonized with *Bifidobacterium breve*, indicating that probiotic administration alone is not sufficient for colonization and subsequent beneficial effects [75]. The effect of probiotics also may be specific to the formulation studied. Because formulations vary, this may decrease the overall signal for probiotic efficacy.

Studies of probiotics in critically ill adults have shown similarly mixed results, but a meta-analysis of probiotics for infection prophylaxis in critically ill patients demonstrated an association between probiotics and reduction in infections and ventilator-associated pneumonia [76]. The included trials were heterogeneous with respect to inclusion criteria and probiotic species used and only a subset specifically included patients with sepsis. Concurrent broad-spectrum antibiotic use in patients with sepsis may limit probiotic colonization and beneficial effects. Furthermore, concerns over probiotic-associated bacteremia, as demonstrated in a recent genomic study [77], have given clinicians pause.

Previous studies have focused largely on probiotics containing *Lactobacillus* and *Bifidobacterium* species, but several other next-generation probiotic species appear promising in pre-clinical studies. *Akkermansia*, for example, is associated with increased sepsis survival in mice [37]; in a separate study, *Akkermansia* directed T cell differentiation [78], providing one mechanism by which *Akkermansia* may improve sepsis outcomes. Probiotic “consortia,” i.e., groups of probiotics, can theoretically be manufactured to achieve specific synergistic effects. For example, one probiotic consortium degraded ampicillin and decreased colonization with vancomycin-resistant *Enterococcus* (VRE) in antibiotic-treated mice [79]; it is plausible that this could decrease subsequent VRE bacteremia. More mechanistic studies are needed to determine the impact of specific next-generation probiotic species and consortia prior to translating these findings into probiotics for patients with or at risk of sepsis.

### Fecal microbiota transplant

While probiotics and synbiotics contain only one or a small number of bacterial species, FMT potentially allows for colonization of an entire donor gut microbiome in a recipient. Therefore, FMT may allow for more robust microbiome reconstitution and impact sepsis outcomes through several mechanisms including SCFA production and immune regulation.

There are five case reports of FMT for non-CDI sepsis [43, 80–82] (Table 2). These reports vary in quality, as some do not specify the suspected cause of sepsis [43, 80, 82]. Prior to FMT receipt, these patients suffered from prolonged ICU admissions with complications including bacteremia, MDR bacterial infection, respiratory failure, and organ dysfunction. Notably, many of the cases had prolonged hemodynamic compromise despite not having a specific defined infection for up to weeks prior to FMT [43, 80, 82]. This may indicate a prolonged immune-dysregulated state associated with sepsis and its numerous consequences.

**Table 2:** Published case reports on the use of fecal microbiota transplant (FMT) to treat sepsis (other than sepsis secondary to *Clostridioides difficile* infection)

Author, year [citation]	Location	Patient age, sex, comorbidity	ICU complications	Sepsis etiology	Gut microbiome changes with FMT	Outcome
Li, 2014 [80]	China	29F, UC	Bacteremia, shock	Unclear; prolonged diarrhea	• Pre: few anaerobes, abundant pathogens including *Enterobacter*	Clinical improvement
					• Post: shifted to donor stool; increased Bacteroides and Firmicutes	
Li, 2015 [43]	China	44F, s/p proximal gastrectomy and vagotomy for NET	Shock, respiratory failure (V-V ECMO), AKI (CRRT)	Unclear; prolonged diarrhea	• Pre: few anaerobes, abundant pathogens including *Enterobacter* and *Klebsiella*	Clinical improvement
					• Post: increased Firmicutes; decreased pathobionts	
Wei, 2016 [82]	China	65M, hemorrhagic CVA	Shock, respiratory failure, bacteremia	Unclear; prolonged diarrhea	• Pre: different from donor	Clinical improvement
					• Post: increased Firmicutes, Bacteroides	
Wei, 2016 [82]	China	84M, ischemic CVA	AKI (CRRT)	Unclear; prolonged diarrhea	• Pre: different from donor	Clinical improvement
					• Post: increased Firmicutes, decreased pathobionts	
Gopalsamy, 2018 [81]	USA	57M, TBI	MDRO infection, respiratory failure	Pneumonia	Not studied	Death

*AKI* acute kidney injury, *CRRT* continuous renal replacement therapy, *CVA* cerebrovascular accident, *F* female, *ECMO* extra-corporeal membrane oxygenation, *FMT* fecal microbiota transplant, *ICU* intensive care unit, *M* male, *MDRO* multi-drug resistant organism, *NET* neuroendocrine tumor, *TBI* traumatic brain injury, *UC* ulcerative colitis, *USA* United States of America, *V-V* veno-venous

In four of five cases, FMT was temporally associated with improved organ function, resolution of sepsis, and survival [43, 80, 82]. Prior to FMT, the patients that survived had gut microbiomes characterized by abundance of hospital-associated pathogenic bacteria. After FMT, the patients’ microbiomes were similar to those of their stool donors and had higher abundance of commensal bacteria [43, 80, 82]. In one case, FMT correlated with lower systemic levels of pro-inflammatory cytokines, although the direct impact of FMT remains uncertain [43].

Although these case reports are intriguing, FMT for sepsis is clearly preliminary. Research to identify patients who may benefit and effect on patient-centered outcomes could inform its utility in broader application for sepsis. Additionally, multiple recent reports of *E. coli* infections acquired via FMT, including several resulting in death, highlight the need for careful donor screening, especially in patients with significant medical comorbidities [83, 84].

## Conclusions and future directions

Gut microbiome disruption appears to be a risk factor for sepsis and subsequent organ dysfunction. The gut microbiome affects host susceptibility and response to sepsis through a number of pathways. Specifically, fewer beneficial taxa allow for pathobiont colonization and alter host immune response and SCFA production. A direct link between abnormal gut microbiome development and sepsis risk has been established in neonates. However, there are several aspects of the relationship between the gut microbiome and sepsis that require further study (Table 3). In adults, there is evidence of a correlation between gut microbiome alteration and sepsis risk; however, the potential confounders require further investigation. Once sepsis develops, the gut microbiome appears to effect end-organ dysfunction including ARDS and delirium, although mechanisms require further validation. Lastly, strategies to alter the gut microbiome, including FMT, either prior to sepsis onset or during the course of sepsis may be beneficial for selected patients with significant change in microbiome structure and function. Before further uptake of FMT in sepsis, several factors including patient selection, timing, and optimal route of administration require further research. Hopefully, improved mechanistic insights into the interaction between the gut microbiome and sepsis will allow for development of novel microbiome-based therapeutics to mitigate sepsis morbidity and mortality.

**Table 3:** Proposed research priorities for study of the interaction between the gut microbiome and sepsis

Current knowledge gap:	Studies should address:
The role of gut microbiome alteration on sepsis predisposition	• Longitudinal microbiome changes in groups at high risk for sepsis
	• Microbiome characteristics that indicate high risk for sepsis, including whether these can be used for prediction/diagnosis
	• Mechanisms linking increase or decrease of specific taxa to sepsis risk
	• How gut microbiome alterations with loss of protective taxa impacts immune dysregulation predisposing to sepsis
	• Impact of SCFAs on protection from sepsis
	• If altering the gut microbiome can decrease sepsis risk
How the gut microbiome impacts sepsis outcomes	• Correlation of markers of gut microbiome alteration with end-organ dysfunction and mortality
	• Whether specific patterns of alteration can predict adverse outcomes in sepsis
	• Gut microbiome changes that contribute to dysregulated immune responses of sepsis
	• Role of pathobionts and antibiotic resistance genes in antibiotic selection
Whether microbiome-directed therapeutics can impact sepsis outcomes	• Which patients with microbiome alteration may benefit from attempting to restore the gut microbiome to lower risk of sepsis
	• Which patients with sepsis may benefit from microbiome-directed therapeutics to improve sepsis outcomes
	• The ideal method of gut microbiome therapeutics (i.e., probiotics, FMT)
	• The specific dose, timing, and frequency of FMT that may benefit patients in these groups

*FMT* fecal microbiota transplant, *SCFA* short-chain fatty acid

## Data Availability

Not applicable

## Abbreviations

AKI: Acute kidney injury
ARDS: Acute respiratory distress syndrome
CDI: *Clostridioides difficile* infection
FMT: Fecal microbiota transplant
ICU: Intensive care unit
LPS: Lipopolysaccharide
MDR: Multi-drug resistant
RCT: Randomized controlled trial
rRNA: Ribosomal RNA [ribonucleic acid]
SCFA: Short-chain fatty acid
SIRS: Systemic inflammatory response syndrome
VRE: Vancomycin-resistant *Enterococcus*
